# Detection of quantitative trait loci for maternal traits using high-density genotypes of Blonde d’Aquitaine beef cattle

**DOI:** 10.1186/s12863-016-0397-y

**Published:** 2016-06-21

**Authors:** Alexis Michenet, Marine Barbat, Romain Saintilan, Eric Venot, Florence Phocas

**Affiliations:** 1 UMR GABI, INRA, AgroParisTech, Université Paris-Saclay, Jouy-en-Josas, 78352 France; 2 AURIVA, Les Nauzes, Soual, 81580 France; 3 ALLICE, 149 rue de Bercy, Paris, 75012 France

**Keywords:** Beef cattle, Calving performance, Suckling performance, Milk yield, Quantitative trait locus (QTL)

## Abstract

**Background:**

The genetic determinism of the calving and suckling performance of beef cows is little known whereas these maternal traits are of major economic importance in beef cattle production systems. This paper aims to identify QTL regions and candidate genes that affect maternal performance traits in the Blonde d’Aquitaine breed. Three calving performance traits were studied: the maternal effect on calving score from field data, the calving score and pelvic opening recorded in station for primiparous cows. Three other traits related to suckling performance were also analysed: the maternal effect on weaning weight from field data, milk yield and the udder swelling score recorded in station for primiparous cows. A total of 2,505 animals were genotyped from various chip densities and imputed in high density chips for 706,791 SNP. The number of genotyped animals with phenotypes ranged from 1,151 to 2,284, depending on the trait considered.

**Results:**

QTL detections were performed using a Bayes C approach. Evidence for a QTL was based on Bayes Factor values. Putative candidate genes were proposed for the QTL with major evidence for one of the six traits and for the QTL shared by at least two of the three traits underlying either calving or suckling performance. Nine candidate genes were proposed for calving performance among the nine highlighted QTL regions. The neuroregulin gene on chromosome 27 was notably identified as a very likely candidate gene for maternal calving performance. As for suckling abilities, seven candidate genes were identified among the 15 highlighted QTL. In particular, the Group-Specific Component gene on chromosome 6, which encodes vitamin D binding protein, is likely to have a major effect on maternal weaning weight in the Blonde d’Aquitaine breed. This gene had already been linked to milk production and clinical mastitis in dairy cattle.

**Conclusion:**

In the near future, these QTL findings and the preliminary proposals of candidate genes which act on the maternal performance of beef cows should help to identify putative causal mutations based on sequence data from different cattle breeds.

**Electronic supplementary material:**

The online version of this article (doi:10.1186/s12863-016-0397-y) contains supplementary material, which is available to authorized users.

## **Background**

Maternal performance traits are of major economic and ethical importance to the sustainable breeding of beef cattle worldwide. Of these traits, suckling performance and calving ease are the essential maternal abilities that must be considered among the breeding objectives of any beef cattle breed [1–4]. Calving difficulty should be limited because it markedly affects the welfare of both the cow and calf and the profitability of herds, because of increased labour and veterinary costs, calf mortality rates and the time before a cow can breed again. The suckling performance of beef cows is also important in order to achieve good calf growth with little or no input of concentrate in the diet.

The only way to assess suckling and calving performance based on field data is by estimating genetic maternal effects on weaning weight and the calving difficulty score, respectively. Finer phenotypes can only be recorded in test stations, and particularly milk yield records using the calf weigh-suckle-weigh technique and pelvic opening measurements after calving. Current developments in molecular biology and statistical methodologies have provided new tools to unravel the genetic determinism of these traits. However, because of the cost and difficulty in recording such phenotypes at a large scale, the literature contains very few studies dedicated to quantitative trait locus (QTL) detection for maternal traits in beef cattle, and these studies are based on field data [5–7]. The first aim of our paper was therefore to analyse the genetic architecture of the maternal traits of beef cows by comparing QTL analyses of field traits and station traits. The second aim was to identify as accurately as possible the principal genomic regions affecting the calving and suckling performance of Blonde d’Aquitaine cows.

## **Methods**

### Genotypes

A total of 2,505 Blonde d’Aquitaine animals (the phenotypes of 909 bulls and 1,596 females were considered during the study) were genotyped using three different DNA chips: the Bovine EuroG10K BeadChip® (customized low density chip), the Bovine SNP50 BeadChip® (MD chip), or the Bovine HD BeadChip® (777 K markers corresponding to a high density (HD) chip). The females were the progeny of 78 sires (20 daughters per sire) evaluated for their maternal traits in a progeny testing station. Most of the females (1,351) were genotyped using the low density chip, the remainder using the MD chip. Most of the bulls were genotyped with the MD chip, but the 282 main genetic ancestors were genotyped with the HD chip. In particular, most of the sires (69 out of 78) of the genotyped females were genotyped using the HD chip. After quality controls that included call rate higher than 90 % and a Hardy-Weinberg equilibrium test (P-value > 10^-4^), 706,791 single nucleotide polymorphisms (SNP) of the HD chip were retained, 37,634 SNP for the 54 K chip and 7,660 for the low density chip. Imputations were performed within the Blonde d’Aquitaine breed in two steps (from low density to MD, then from MD to HD). A total of 2690 MD genotypes (Additional file 1) were used to impute the female genotypes from low density to MD. The HD genotypes of 325 Blonde d’Aquitaine main ancestors were used for the imputation from MD to HD. BEAGLE 3.3.0 software was used in both cases for the imputation [8]. Hozé et al. [9] provided a detailed description of these genotype editing and imputation procedures. The SNP were mapped to the UMD 3.1 bovine genome sequence assembled by the Center of Bioinformatics and Computational Biology at the University of Maryland (US).

### Phenotypes recorded in the field

The French national genetic evaluation process provided a large dataset of on-farm records for calving and suckling performance in the Blonde d’Aquitaine breed [10] for both males and females. Estimated breeding values (EBV) were derived from a best linear unbiased predictor (BLUP) animal model with maternal effects. Deregressed Estimated Breeding Values (DEBV) were computed to obtain “pseudo-phenotypes” for each animal, according to the methodology proposed by Garrick et al. [11].

The evaluation of calving difficulty was based on the maternal genetic effect for the birth condition score recorded at the calving of females or at the calving of the female progeny of bulls. This score was allocated by the farmer and ranged from 1 (no assistance) to 4 (caesarian section). In the Blonde d’Aquitaine breed, most calvings (77 %) were unassisted (score 1), 17 % experienced minor difficulties requiring some assistance (score 2), 4 % were mechanically assisted (score 3) and 2 % involved caesarian sections (score 4). For primiparous cows, calving difficulties were more common, with 4 % of caesarian sections, 8 % involving considerable assistance and only 62 % of unassisted calvings [12]. The evaluation of suckling performance was based on the maternal genetic effect for the weaning weight (WWm) of the calf. The weaning weight was adjusted at 210-days: the average performance was 298 kg and 273 kg, respectively, for male and female calves born during the 2013 birth campaign [12].

The category, origin, heritability and number of animals genotyped with their performance are shown in Table 1. The heritability of these maternal traits was low. The on-farm population comprised two types of animals, females and bulls. The females had only a few progeny records (between one and five) and poor DEBV reliability (0.05 and 0.08 for CSm and WWm, respectively), with 1,377 animals for CSm and 706 for WWm. The bull population comprised a large population of young or service sires with low to high DEBV reliabilities (0.29 and 0.33 for CSm and WWm, respectively). Of these, field information was available on 907 animals relative to CSm and 725 for WWm.

**Table 1:** Definition of traits (category and origin of performance, heritability) and number of phenotypes

Trait	Category	Origin	Heritability	Number of animal with phenotypes and genotype
Calving difficulty score (CS)	Calving	Station	0.44	1,250
Pelvic opening (PO)	Calving	Station	0.37	1,239
Maternal effect on calving difficulty score (CSm)	Calving	Farm	0.04	2,284
Milk yield (MY)	Suckling	Station	0.35	1,151
Udder swelling score (US)	Suckling	Station	0.48	1,250
Maternal effect on weaning weight (WWm)	Suckling	Farm	0.10	1,431

### Phenotypes recorded in station

The station data consisted in the different phenotypes of 1,250 females born between 2002 and 2012 corresponding to the progeny of 78 genotyped sires that had been progeny tested for maternal traits in the context of the French Blonde d’Aquitaine breeding programme. Direct and maternal genetic effects could not be dissociated in the progeny test evaluation because only the first calf was recorded for the daughters and they were bred from different dams with unknown pedigree.

In the test station, two traits were recorded with respect to calving performance. Firstly, the calving difficulty score (CS) was recorded in the same way as for the field data. However, the station females were only primiparous cows that calved younger (around 26 months old) than on commercial farms (around 35 months old) and they were mated with a non-easy calving bull in order to better discriminate sires with respect to the calving ease of their daughters. Thus the CS distribution differed critically from that of the field data: 29 % of unassisted calvings, 42 % with slight assistance, 16 % mechanically assisted and 13 % of caesarian sections. Secondly, cow pelvic opening (PO) was measured one week after calving. This corresponds to the area estimated by the product of the median bi-iliac width by the sacro-pubian height, the average area being 0.0307 m^2^ with a standard deviation of 0.0043 m^2^. The genetic correlation between CS and PO was estimated at 0.50. Concerning suckling performance, two traits were considered in the test station. Firstly, suckling performance was assessed by the calf weigh-suckle-weigh technique to obtain an estimate of milk yield (MY). Measurements were performed in the morning and evening at the 60th and 120th days after calving. MY was estimated from the weighted average of the 60th-day measurement and 120th-day measurement, with respective weightings of one-third and two-thirds. The average MY was 5.54 kg, with a standard deviation of 1.39 kg. Another trait that appeared to be a potential indirect predictor of suckling performance was the udder swelling score (US). A five-point scale was used to assess the degree of udder swelling before calving, with 1 and 5 points corresponding to the least swollen and most swollen udder, respectively. The US distribution of the score was mainly split around the three central scores (2: 29 %, 3: 42 %, 4: 23 %), with only 1 % of udders being scored 1 and 5 % scored 5. The estimated genetic correlation between MY and US is 0.60.

For the analysis, calving performance was studied considering by pooling the field phenotype CSm with the station traits CS and PO; suckling performance was analysed based on the field trait WWm and the station traits MY and US. A large proportion of the females whose first calving had been recorded in station had their other calvings recorded on farm: 1,031 females had phenotypes for CS, PO and CSm, and 561 females had phenotypes for MY, US and WWm.

### Statistical model

QTL detections were performed using observed and imputed HD genotypes. The QTL detections for station traits were based on individual performance corrected for the fixed environmental effects used in the genetic evaluation described by Phocas and Sapa [13]. For all station traits, these environmental effects consisted in the birth region and birth year-period of the heifers, the calving parity of their dam and the age at calving of the heifers (fitted as a covariable). With respect to calving performance traits, the calving period within year and the calf sex were also added to the previous model. For MY, the suckling batch within year was included in the model rather than the calving period within year.

Regarding QTL detections for field traits, DEBV data from the national on-farm evaluation were used in a weighted analysis with the corresponding weightings depending on DEBV reliabilities, according to the methodology proposed by Garrick et al. [11]. This method aims to remove parental contributions from EBV so as to account only for individual and progeny performance in the DEBV and the associated weightings.

Because the number of SNP effects to be estimated was far higher than the number of records, QTL detections were based on a Bayesian variable selection approach in order to resolve this statistical challenge [14]. We considered a BayesC strategy [15] where a fraction of SNP, π, was assumed to have a non-zero effect at each iteration. SNP estimates were made using a mixture of π marker proportion with a normal effect distribution N(0, σ^2^_*a*_) and 1- π marker proportion with a mass point at 0. The general linear mixed model was defined as in equation:

$$ {y}_i=\mu + {\displaystyle \sum_{j=1}^n}{z}_{ij}{a}_j{\delta}_j + {e}_i $$

where y_i_ is the phenotype of the animal *i*, μ the mean for the considered trait, *n* the number of SNP, z_ij_ the genotype at locus *j* for animal *i* (with z_ij_ = 1 for the homozygote with allele 1 at locus *j*, z_ij_ = -1, with the opposite and z_ij_ = 0 for the heterozygote), *a*_j_ the effect of the marker *j*, *δ*_j_ the indicator variable (*δ*_j_ =1 if marker *j* is selected at a given iteration, *δ*_j_ =0 otherwise), and *e*_i_ the random residual effect. We used GS3 software [16] to perform these analyses.

A total of 100,000 iterations were performed, with a burn-in of 20,000 iterations. We used a polygenic BLUP model to obtain a preliminary estimate of genetic variance (σ^2^_*u*_) and residual variance (σ^2^_*e*_).

The total additive genetic variance captured by the markers was computed as follows [17]:

$$ \widehat{\sigma_u^2}=2\ {\displaystyle \sum_{i=1}^n}\ {p}_i\left(1-{p}_i\right)\ \widehat{\sigma_a^2} $$

where *p*_*i*_ is the frequency of allele *i* and σ^2^_*a*_ is the marker variance. The π value retained was 0.025 %, corresponding to 177 SNP selected at each iteration from markers on the HD chip. According to the traits analysed, 63 to 97 % of the total genetic variance was captured by the markers. The inclusion of a polygenic component in a model accounting for 40 % of σ^2^_*u*_ was tested in order to capture a larger share of the genetic variance and to directly account for pedigree relationships across animals. The results showed that the same QTL were detected and located at the same positions using the two models (with or without a polygenic component), meaning that the polygenic component was not useful to prevent any misleading results due to stratification of the population. Only minor changes were detected in the degree of evidence of the QTL, because of the sensitivity of the Bayesian factor to the prior distribution of model parameters [18]. Therefore, the parsimonious model (without the inclusion of a polygenic component) was retained when presenting the results.

### Definition of QTL regions

The degree of association between each SNP and the different phenotypes was assessed using the Bayes Factor (BF) [19]. The BF involves π and P_*i*_, the probability of the SNP having a non-zero effect, as in equation [20]:

$$ \mathrm{B}\mathrm{F} = \frac{\raisebox{1ex}{${\mathrm{P}}_{\mathrm{i}}$}\!\left/ \!\raisebox{-1ex}{$\left(1-{\mathrm{P}}_{\mathrm{i}}\right)$}\right.}{\raisebox{1ex}{$\uppi $}\!\left/ \!\raisebox{-1ex}{$\left(1-\uppi \right)$}\right.} $$

The Bayes factor offers a clear and rigorous framework to compare competing models. It is the recommended statistical criterion to be considered when using the Bayesian method to detect QTL [21, 22]. Classical hypothesis tests try to discard the null hypothesis in favour of an alternative hypothesis, while BF provides a ratio of probabilities between models, without it being necessary to define the null or alternative model. Because its results can be expressed in terms of an increase from prior to posterior probabilities of the SNP being “in” the model [20, 23] it is no longer necessary to calculate significance levels, with either simulations or theoretical approximations. The Bayes factor is not dependent on asymptotic properties and can be used safely, even with small samples [23].

**Fig. 1:**
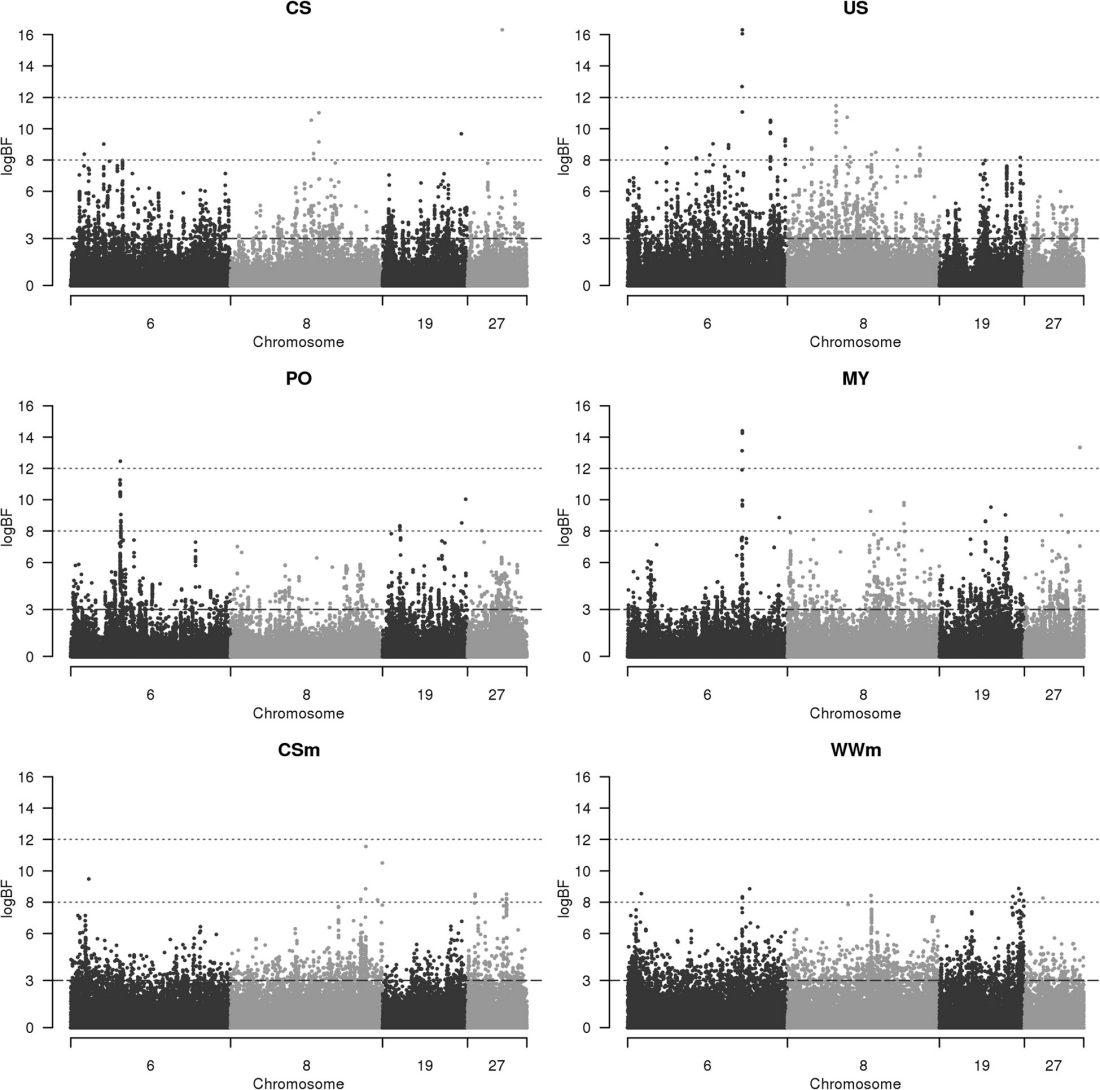
Genome-wide plots of chromosomes 6, 8, 19 and 27 for the six traits. Traits affecting calving performance in the first column: calving difficulty score (CS), pelvic opening (PO) and maternal effect on calving difficulty score (CSm). Traits affecting suckling performance in the second column: milk yield (MY), udder swelling score (US) and maternal effect on weaning weight (WWm)

A transformation of the BF (logBF) was considered in order to obtain a clearer visual appraisal of all QTL regions at the chromosome scale (see Fig. 1). LogBF was computed as twice the natural logarithm of the BF. This logarithmic scale produced values within the same usual range as deviance and likelihood ratio test values, thus facilitating the determination of thresholds to define QTL as proposed by Kass and Raftery [20]. These authors suggested the following categories to classify the strength of the evidence provided by logBF: evidence in favour of the hypothesis is considered to be very strong for values >10, strong for values between 6 and 10 and positive for values between 2 and 6. Because an SNP with a higher logBF may not necessarily correspond to that closest to the causal mutation, SNP with logBF >3 located close to the peak SNP were also included in the QTL region when these SNP lay within a sliding window of 0.5 Mb on either side of the peak SNP. Sliding the window was performed when at least one SNP with logBF >3 was found in the current window. The start and end bounds of the QTL regions were defined by the locations of the last SNP with logBF >3 that were integrated in the QTL region. The corresponding final intervals of all QTL regions are shown in Additional file 2 for the six traits under analysis.

### Comparison of QTL regions

The detection of QTL for the three traits grouped under the same performance characteristic, either ‘calving performance’ or ‘suckling performance’, were compared at a threshold of logBF above 8, in order to better assess the relevance of each QTL detected to the corresponding maternal performance. Aware of the high risk of detecting false positive QTL with limited data [24], we believe that searching for causal mutations would be more efficient by focusing either on QTL detected with major evidence (logBF >12) for a single trait or on QTL detected (logBF >8) for two or three of the traits within the same performance characteristic. The comparative analysis of QTL detections regarding different traits related to the same performance characteristic offered good insurance against false positive results.

**Fig. 2:**
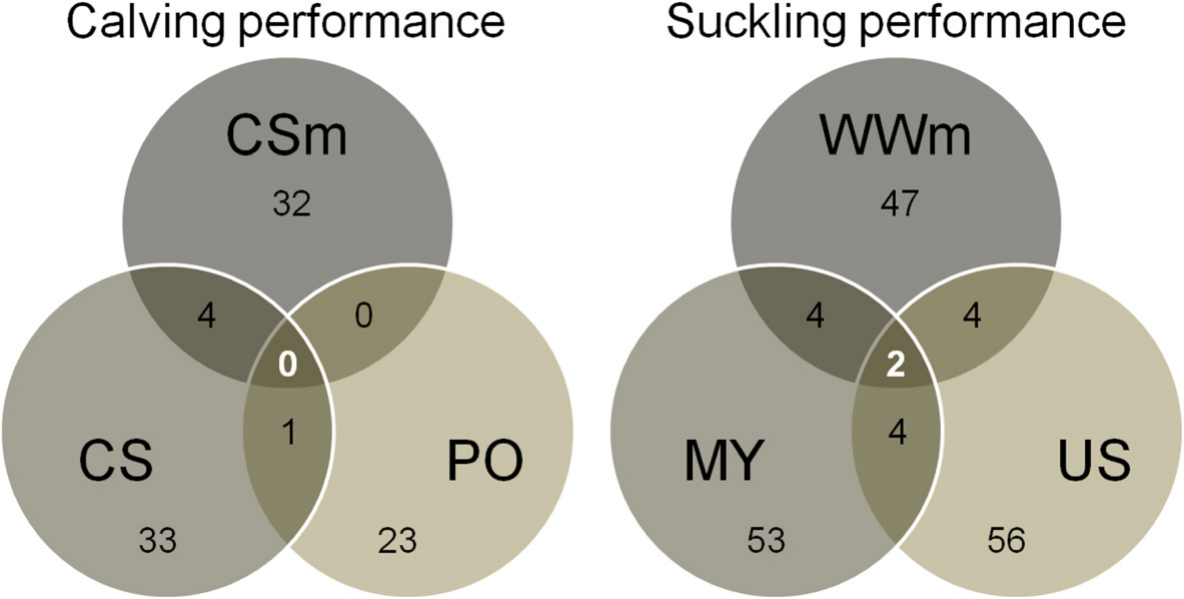
Venn diagram showing common QTL between calving and suckling performance traits. Calving performance: difficulty score (CS), pelvic opening (PO), maternal effect on calving difficulty score (CSm). Suckling performance: milk yield (MY), udder swelling score (US) and maternal effect on weaning weight (WWm)

During our study, the field data and station data were recorded completely independently, but concerned animal populations that were closely related. Venn diagrams were built to illustrate the numbers of QTL detected for each trait and the numbers that were common to two or three traits (Fig. 2). QTL with major evidence for each trait and QTL common to different traits were studied in more detail and compared with findings in the literature, mainly drawn from the Animal QTL database (www.animalgenome.org/cgi-bin/QTLdb/index) [25]. Some candidate genes were proposed using Ensembl (ensembl.org).

## Results

For all traits, QTL characteristics (peak location, peak logBF and region size) are reported in Additional file 2.The average sizes of QTL regions were in average between 0.7 and 1.1 Mb for station traits, whereas they were between 2.1 and 2.4 Mb wide for field traits. Table 2 shows the QTL with major evidence.

**Table 2:** Major QTL (logBF >12) locations detected for calving and suckling performance

Trait	Chromosome	Start - end position	Peak position	peak logBF
PO	6	38.287 - 39.552 Mb	38.955154 Mb	12.5
CSm	11	70.279 - 73.525 Mb	72.949696 Mb	12.2
CS	21	39.493 - 40.269 Mb	39.668783 Mb	12.9
CS	27	27.504 - 27.877 Mb	27.813590 Mb	16.3
CSm	28	15.064 - 15.531 Mb	15.458787 Mb	13.6
US	6	88.485 - 88.959 Mb	88.922396 Mb	16.3
MY	5	28.577 - 29.137 Mb	29.072132 Mb	13.4
MY	6	88.485 - 89.223 Mb	88.919352 Mb	14.4
MY	10	69.747 - 72.705 Mb	70.306697 Mb	12.8
MY	13	82.728 - 84.013 Mb	83.805618 Mb	12.1
MY	20	3.861 - 7.327 Mb	5.504819 Mb	13.2
MY	27	42.375 - 43.266 Mb	42.896895 Mb	13.4
WWm	7	24.793 - 25.722 Mb	25.004920 Mb	13.9
WWm	28	19.766 - 20.801 Mb	19.922560 Mb	14.6
WWm	28	29.412 - 29.772 Mb	29.570491 Mb	12.1

### Description of QTL underlying the calving performance of beef cows

For calving performance, between 23 and 33 QTL were detected with strong evidence for each trait (Fig. 2). A total of five QTL were detected with major evidence for calving performance, two for CS, one for PO and two for CSm. Considering the CSm field trait as a reference, two-by-two comparisons of the three QTL detections were plotted on Fig. 2. No QTL were evidenced as being common to the three traits. Among the 32 QTL detected for CSm, four shared a common region with the QTL for CS, but no QTL for CSm shared a common region with any of the QTL for PO. Only one QTL was detected for both CS and PO. Comparing QTL region intervals for common QTL made it possible to limit the likely region for the QTL by only considering regions overlapping across the detections. For the five common QTL, the average common region size was 0.387 Mb while the average size of the original regions was 1.612 Mb.

A single QTL with major evidence was detected for PO on chromosome 6 between 38.287 Mb and 39.552 Mb (Table 2) and is plotted in Fig. 1. This region was highlighted as containing eight genes, which in particular included *LAP3, NCAPG* and *LCORL* (see Table 4)*.*

Concerning CS, the main QTL was detected on chromosome 27 with a peak position at 27.814 Mb (Table 2). The same region was also detected for CSm, as can be seen in Fig. 1. The common region between CS and CSm QTL contained a single gene: *NRG1* (Table 4). The second QTL with major evidence for CS corresponded to a peak position at 39.669 Mb on chromosome 21 (Table 2) and was located 0.01 Mb from a pseudogene that has not yet been identified in bovine species. However, the sequence of the *FOXG1* gene (Table 4), which was the only gene in the QTL, was aligned in human, mouse and rat in this region (and in this region only).

The common QTL for CS and PO was evidenced on chromosome 19 with a very small common region (<0.1 Mb; Table 3). In addition, this region was also detected as a putative QTL for CSm, as shown in Fig. 1. However, no mapped gene or structural CNV was found in this region.

**Table 3:** Common QTL locations and peak positions identified for calving and suckling performance

Traits	Chromosome	Common region	Peak position trait 1	Peak position trait 2
CSm - CS	1	0.413 - 0.884 Mb	3.40862	0.575063
CSm - CS	6	12.391 - 12.937 Mb	15.164578	12.391211
PO - CS	19	60.053 - 60.141 Mb	60.369264	60.140763
CSm - CS	25	13.798 - 14.530 Mb	16.085565	14.223691
CSm - CS	27	27.775 - 27.877 Mb	27.790463	27.81359
US - MY	4	44.177 - 44.917 Mb	44.260073	44.198598
WWm - US	6	88.485 - 88.959 Mb	88.958116	88.922396
US - MY	6	88.485 - 88.959 Mb	88.922396	88.919352
WWm - MY	6	88.485 - 89.223 Mb	88.958116	88.919352
WWm - US	8	60.296 - 61.521 Mb	60.762241	61.044151
US - MY	8	60.348 - 60.353 Mb	61.044151	60.352572
WWm - MY	8	60.348 - 60.353 Mb	60.762241	60.352572
WWm - US	18	33.562 - 34.342 Mb	33.031008	34.538807
WWm - US	19	61.166 - 61.845 Mb	60.504374	61.534509
WWm - MY	20	5.554 - 7.327 Mb	6.392965	5.504819
WWm - MY	20	57.350 - 59.102 Mb	58.801089	58.162729
US - MY	28	43.511 - 44.630 Mb	43.242413	44.036312

Two QTL were detected with major evidence for CSm. A large region was detected on chromosome 11 between 70.279 Mb and 73.525 Mb, containing tens of genes. However, no information was able to confirm this region as a likely QTL for calving performance. A smaller region (<0.5 Mb) was detected on chromosome 28 (Table 2) with the peak SNP included in the *SLC16A9* gene (Table 4).

**Table 4:** Candidate gene symbols, names and positions for calving and suckling performance

Gene symbol	Gene name	Chr^a^	Position	Traits involved
ARSJ	Arylsulfatase Family, Member J	6	12.720022 - 12.803721	CS, CSm
LAP3	Leucine Aminopeptidase 3	6	38.574590 - 38.600027	PO
NCAPG	Non-SMC Condensin I Complex, Subunit G	6	38.765969 - 38.812051	PO
LCORL	Ligand Dependent Nuclear Receptor Corepressor-Like	6	38.840894 - 38.992112	PO
FOXG1	Forkhead Box G1	21	39.655563 - 39.657038	CS
NDE1	nudE Neurodevelopment Protein 1	25	14.189695 - 14.237237	CS, CSm
MYH11	Myosin 11	25	14.218281 - 14.343745	CS, CSm
NRG1	Neuroregulin 1	27	27.624065 - 27.702831	CS, CSm
SLC16A9	Solute carrier family 16, member 9	28	15.428478 - 15.474740	CSm
GC	Group-Specific Component	6	88.695940 - 88.739180	WWm, MY, US
SLC11A2	Solute carrier family 11, member 2	5	29.012107 - 29.034478	MY
RGP1	Retrograde Golgi Transport Homolog	8	60.328033 - 60.333812	WWm, US, MY
KCNJ2	Potassium channel, inwardly rectifying subfamily J, member 2	19	61.185603 - 61.195897	WWm, US
KCNJ16	Potassium channel, inwardly rectifying subfamily J, member 16	19	61.226690 - 61.229571	WWm, US
TRIO	Trio Rho Guanine Nucleotide Exchange Factor	20	58.714145 - 58.945942	WWm, MY
ANXA7	Annexin A7	28	29.552050 - 29.573976	WWm

^a^Chromosome

At the beginning of chromosome 1, a large QTL region (>6 Mb) was detected for CSm and also for CS; the common QTL region (Table 3) was shorter (<0.5 Mb) but still contained six genes. The common QTL region on chromosome 6 for CS and CSm (Table 3) included the CS peak and contained a single gene: Arylsulfatase Family, Member J (*ARSJ*).

A common QTL was detected for CS and CSm on chromosome 25, with the CS peak included in the common region (Table 3) where ten genes were mapped. Two genes with opposite strands overlapped at this peak location: *NDE1* gene (Table 4) in the forward strand and *Myosin 11* (*MYH11*) in the reverse strand.

### Description of QTL underlying the suckling performance of beef cows

As for suckling performance, the number of QTL detected with strong evidence was a slightly higher than for calving performance: ranging from 47 QTL for WWm to 56 for US. Considering the field trait WWm as a reference, two two-by-two comparisons of the three QTL detections were plotted on Fig. 2. Among the 47 QTL detected for WWm, only two QTL were common to the three traits, while two other QTL shared a common region with QTL for MY and two QTL a common part with the QTL for US. Two final QTL were common to MY and US. For the eight common regions, the average common region size was 0.915 Mb, while the average size of the original regions was 2.447 Mb. A total of ten QTL were detected with major evidence for suckling performance: six for MY, three for WWm and one for US.

Regarding the three QTL detected with major evidence for WWm, two QTL were detected on chromosome 28; the second region detected on chromosome 28 contained a total of eight genes. The peak SNP (29.570 Mb) was included in the *Annexin A7* (*ANXA7*) gene (Table 4).

**Fig. 3:**
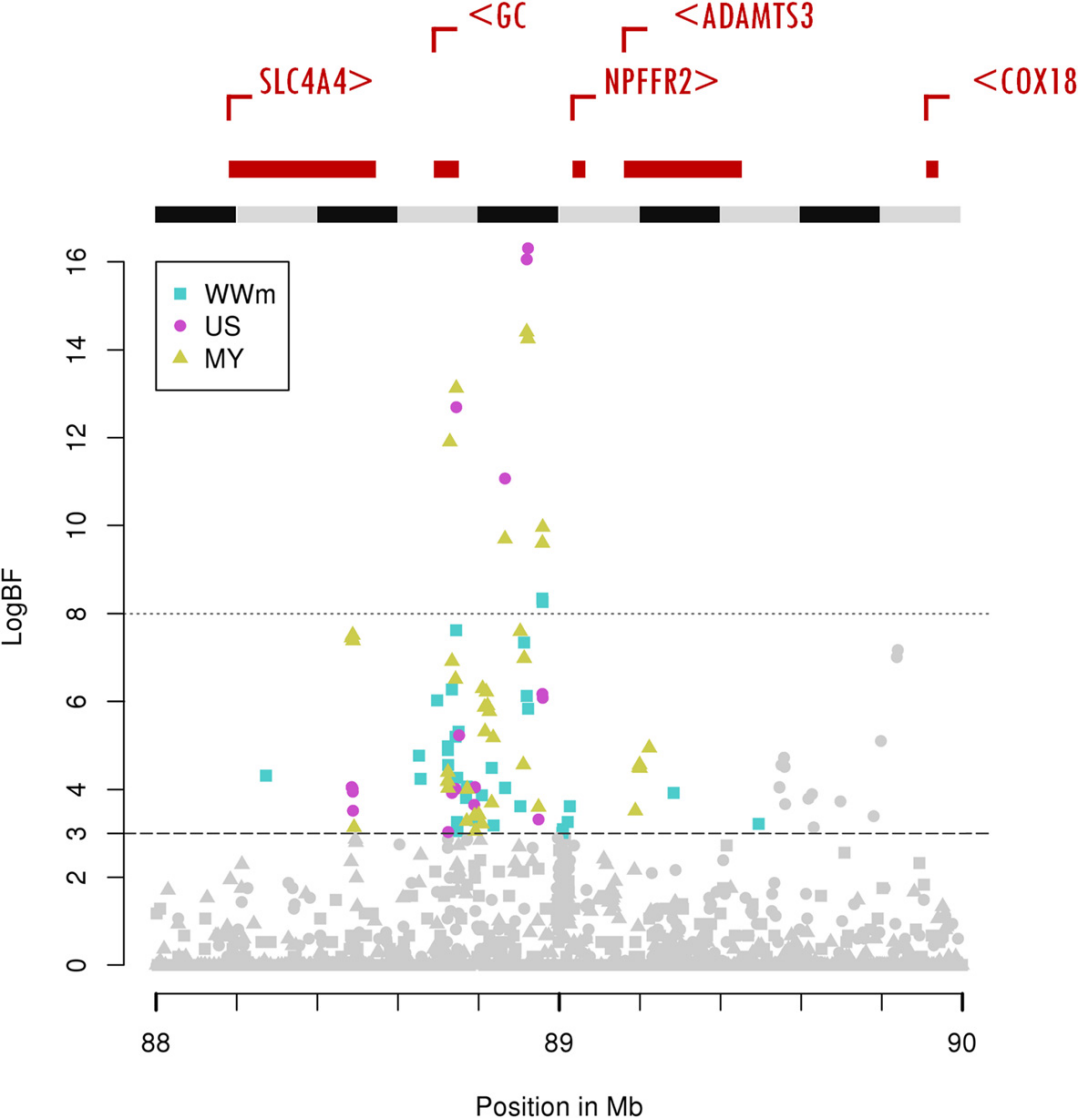
Plot of logBF for suckling traits on chromosome 6 between 88 and 90 Mb. The three suckling traits are maternal effect on weaning weight (WWm), udder swelling score (US) and milk yield (MY). The known genes from Ensembl (ensembl.org) are plotted at the top of the Figure. LogBF: twice the natural logarithm of Bayes Factor; Mb: mega base pairs

The main QTL for US (logBF = 16.3) was located on chromosome 6. This region was also detected as a QTL with major evidence for MY (logBF = 14.4) and with strong evidence for WWm (Fig. 1). Fig. 3 offers a zoom on the plots for the three detections relative to suckling performance in the window spanning 88 Mb to 90 Mb on chromosome 6, with the known genes added at the top of the Figure. As can be seen from Fig. 3, peak SNP for US and MY detections were nearby (distance <0.003 Mb). The linkage disequilibrium between these two peak markers was almost complete (r^2^ > 0.99) among the genotyped females. This region was around 1 Mb after the casein cluster of genes (*CSN1S1*, *CSN1S2*, *CSN2* and *CSN3*) that affect casein milk composition and hence milk protein content [26]. No QTL was detected in our study for suckling traits in this casein cluster region which spans from 87.141 Mb to 87.393 Mb. As seen in Fig. 3, our peak markers were found in an intergenic region between the *GC* and *NPFFR2* genes (Table 4). However, an in-depth study of the region showed that QTL regions of the three traits all integrated two different peak SNP (88.745 Mb and 88.922 Mb). These SNP were 0.177 Mb distant and in very high linkage disequilibrium (r^2^ > 0.95). The second peak was located very close to the start of the reverse strand *GC* gene (Table 4).

In addition to this QTL region close to the *GC* gene, five other QTL were detected with major evidence for MY, on chromosomes 5, 10, 13, 20 and 27 (Table 2). The QTL on chromosome 5 was also detected as a putative QTL for WWm. This region was very rich in genes (13 in total, over 0.560 Mb). Combination with the corresponding QTL for WWm enabled a reduction in the likely region to an area between 28.834 and 29.136 Mb, restricting the number of potential genes to six. This short region included the MY peak and several sequence alignments of the *SLC11A2* gene.

On chromosome 8, a very small common region (Table 3) was shared by QTL detected for the three suckling traits (Fig. 1). The MY peak SNP was included in this region, but no gene was mapped to this small common region. The *RGP1* gene (Table 4) was one of the 13 genes included in the common US-WWm region. Moreover, *RGP1* was the gene closest to the small common region (0.02 Mb) shared by the QTL detected for the three suckling traits.

On chromosome 4, a common QTL region was identified for MY and US (Table 3) and contained 12 genes. On chromosome 28, another QTL was detected for both MY and US (Table 3). A total of 19 genes were mapped to this region.

Regarding the common QTL for US and WWm, the region detected on chromosome 18 (Table 3) did not correspond to any obvious candidate genes linked to suckling performance. On chromosome 19, a common region was detected for US and WWm (Table 3) and included two genes: *KCNJ2* and *KCNJ16* (Table 4).

On chromosome 20, two large common QTL regions were detected for WWm and MY: the former at the beginning of the chromosome (5.554 - 7.327 Mb), and the second from 57.350 Mb to 59.102 Mb. For the second region on chromosome 20, the WWm peak SNP (Table 3) was included in the *TRIO* gene (Table 4).

## Discussion

### Definition of QTL regions

Defining QTL regions is critical when trying to compare detection findings relative to different traits. On a simulation dataset, Shurink et al. [19] established a correspondence between high Bayes Factor values and the existence of QTL: the higher the peak logBF, the lower the risk of detecting a false positive QTL. They considered logBF > 10 as being indicative of strong evidence for a QTL, while values between 3.2 and 10 could be considered as putative QTL. Purfield et al. [7] considered varying the threshold for BF in order to define a strong association with a minimum value of 40 (logBF = 7.3) for a very low heritability trait such as perinatal mortality, and of 200 (logBF = 10.6) for a calving ease trait. Legarra et al. [21] considered a threshold on the BF scale of 150, corresponding to a logBF of 10.0 in the natural logarithmic scale.

Based on these previous proposals, we decided to qualify as a QTL a chromosomal region where an SNP with a peak logBF was above a threshold of 8 (BF ≈ 55), and to determine as major evidence when the peak logBF was above a threshold of 12 (BF ≈ 400). In addition, we considered as “putative QTL” a chromosomal region where at least one SNP had a logBF value between 6 and 8. Although Kass and Raftery [20] considered that a logBF value within this interval corresponded to strong evidence for an association, we considered that such evidence was somewhat speculative because we observed that the proportion of post-burn-in iterations that included the corresponding putative QTL regions (as defined below) was always lower than 50 %.

It should be noted that there was a strong correlation (r >0.98) between the estimates of P_*i*_ (underlying the BF) and the absolute value of the estimated effects of each SNP. Therefore, a QTL with major evidence based on the BF value was likely to be a QTL with a major effect on performance.

### QTL comparisons between field and station traits

The proportion of QTL common to the three traits related to the same maternal performance was low, even when only the two station traits recorded on genotyped females either for calving or for suckling performance were considered. This could be partly explained by the moderate genetic correlations between traits related to the same maternal performance; for instance, 0.50 between OP and CS with respect to calving performance and 0.60 between US and MY for suckling performance.

Another explanation was a likely lack of power (due to an insufficient number of genotyped animals with phenotypes) to enable the detection of QTL with minor effects that might explain a significant part of the covariation between traits. In addition, when comparing the results from the two station traits and associated field trait, three other reasons could explain the lack of common QTL between these traits. Firstly, although some of the station population (all 78 sires, 49 % of females for MY and 82 % for CS) also displayed some performance in the field, a large part of the field population differed from the station population. Secondly, for females displaying performance in both cases, a parity effect on maternal traits could result in weak correlations between the first parity performance recorded in station and the field performance for second and subsequent parities. Thirdly, the pseudo-phenotypes used to analyse the field data were DEBV with accuracy that varied considerably from one genotyped animal to another, depending on the number of phenotyped daughters for the sires or the number of phenotyped calves for the females. This resulted in QTL detections that could be strongly influenced by the largest sire families because of their high DEBV weighting. Nevertheless, these large sire families with field performance mainly comprised the sires that were progeny tested in station. It should be noted that the large half-sib population structure did not facilitate the fine mapping of QTL signals.

A comparison of QTL detections between field and station traits enabled the identification of QTL. The risk of false positive results was very limited when a QTL was detected for at least two of the three traits related to the same maternal performance.

In addition, the common region shared by such a QTL across traits was markedly reduced when compared to the initial QTL regions defined for each trait, thus limiting the genomic region requiring a search for candidate genes.

### Identification of genes associated with calving performance

In terms of the QTL region affecting CSm on chromosome 28 (Table 2), Maltecca et al. [27] identified this region as influencing gestation length in Holstein cows. According to these authors, the QTL was not associated with direct calving ease or calf survival. A shorter gestation length is well known to be linked to smaller calf birth weight due to a maternal effect, which means that genes affecting gestation length may have an impact on CSm. In addition, a QTL was detected in the same region for a maternal effect on the birth weight of Blonde d’Aquitaine calves (M. Barbat, personal communication). Our peak marker for this QTL was located within the *SLC16A9* gene (Table 4) which is involved in the transport of monocarboxylates.

The two peaks locations of the QTL detected on chromosome 27 for CS and CSm (Table 3) were very close together (distance <0.025 Mb), thus testifying to the influence of this region on the maternal calving performance of Blonde d’Aquitaine cows. No correspondence regarding QTL was found with the recent study by Purfield et al. [7] which was based on high-density genotypes in dairy and beef cattle. However, Ashwell et al. [28]) reported a corresponding QTL region (between 26.994 Mb and 32.725 Mb) for a direct effect on calving difficulty in Holstein-Friesian cows. It is possible that the QTL have a pleiotropic effect on calving ease via both maternal and direct effects.

The *NRG1* gene identified in this QTL region encodes the neuregulin protein that is produced in numerous isoforms by alternative splicing, thus allowing it to perform a wide variety of functions [29] that are essential to normal development of the nervous system and heart [30]. In particular, an over-expression of *NRG1* in transgenic mice [31] led to changes in dopamine metabolism. Stefos et al. [32] showed that dopamine inhibited prolactin secretion both during labour and post-partum. Another dopamine function was related to the production of stress hormones such as adrenaline. Hydbring et al. [33] showed that adrenaline levels in the blood rose after calving, and this increase was more marked in heifers that required assistance with calving.

The second major QTL for CS on chromosome 21 (Table 2) contained the *FOXG1* gene that affects brain development in humans. *FOXG1* syndrome is rare and has been described as involving impaired development and structural brain abnormalities in infants. Affected children are small at birth and suffer from microcephaly in early childhood [34]. The QTL was not detected for CSm or PO but a QTL for the birth weight of calves born in station was detected in the same region with the same peak SNP (A. Michenet, unpublished results). The QTL thus identified was therefore more likely to be linked to direct effects than maternal CS effects..

The QTL detected on chromosome 6 (Table 2 and Table 3) for PO contained three genes (*LAP3, NCAPG* and *LCORL)* which affect direct calving ease through an impact on animal development and birth weight [6, 35]. The same region was also highlighted as the main QTL across the entire genome for Blonde d’Aquitaine female growth traits (A. Michenet, unpublished results). In addition, the estimated genetic correlation between PO and cow weight at calving was 0.71 based on the station data (A. Michenet, unpublished results). Therefore, this QTL for PO on chromosome 6 could be expected to play a role on cow size rather than on maternal calving performance itself.

The common CS and CSm region on chromosome 6 (Table 3) contained the *ARSJ* gene, which encodes an enzyme related to embryonic development (Ratzka et al. [36]). Another common region for these traits was identified on chromosome 25 (Table 3) with two genes: *NDE1* encoding a protein linked to neuron development and *MYH11* encoding a protein expressed in smooth muscles.

### Identification of genes associated with suckling performance

As for suckling performance, the QTL for WWm detected on chromosome 7 (Table 2) had previously been detected by Sodeland et al. [37] for clinical mastitis in Norwegian Red cattle. The two other QTL for WWm were both detected on chromosome 28 (Table 2) and have not previously been reported in the literature. The second region on chromosome 28 (Table 2) was associated with the *ANXA7* gene which is involved in calcium transport. Martinez-Royo et al. [38] founded a significant association between the *ANXA9* gene (an important paralog of *ANXA7* on chromosome 3) with milk-fat yield in the Holstein breed. Considering the fact that WWm reflects a dam’s ability to induce calf growth through both the quantity and quality of milk, *ANXA7* is a good candidate gene that may affect milk quality.

Among the six QTL detected with major evidence for MY, no correspondence was found with the recent study by Saatchi et al. [6] which was based on 50 K SNP and considered ten different beef cattle breeds. However, McClure et al. [5] used microsatellite markers to detect two of these QTL as affecting maternal weaning weight in Angus cattle. In our analysis, the first QTL region spanned from 82.728 to 84.013 Mb on chromosome 13 (Table 2) and the second spanned from 42.375 to 43.266 Mb on chromosome 27 (Table 2). Two large QTL regions with major evidence for MY were detected on chromosomes 10 and 20 (Table 2) without any regions being common with data in the literature or with QTL for WWm or US in our study.

On chromosome 5, the QTL detected for MY was considered to be major (Table 2) and for WWm to be putative, and contained the *SLC11A2* gene. This divalent metal transporter protein is involved in regulating essential nutrients in milk such as iron [39]. Because of this role, *SLC11A2* is a good candidate gene for a QTL affecting milk production.

Several papers in the literature have mentioned the region detected for the three suckling traits on chromosome 6. When studying milk yield relative to 8,000 Holstein and Jersey bulls, Goddard et al. [40] detected a non-coding SNP located at 88.741 Mb using imputed sequence data. These findings confirmed the importance of the region upstream from the *CG* gene. This gene encodes vitamin D-binding protein, which transports and delivers vitamin D, which is important to blood calcium homeostasis. It is synthesized in the liver and secreted into milk via the circulatory system, with low levels in mature milk but higher concentrations in colostrum [41]. Milk production requires large quantities of calcium, and a deregulation of calcium homeostasis will reduce milk production and lead to milk fever [42]. This mechanism may explain the relationship between vitamin D-binding protein (*GC* gene) and milk production. In addition, Sahana et al. [43] detected a QTL in the same region for clinical mastitis in dairy cows, and also proposed *GC* as a candidate gene. Clinical mastitis affects beef cows at a high prevalence that ranges from 26 to 54 % depending on the period of lactation and breed [44]. These authors highlighted the negative impact of cow mastitis on calf weaning weight. Moreover, Persson Waller et al. [45] noted that beef cows with funnel-shaped teats or pendulous udders had a greater risk of mastitis, although they did not find any association between udder health and calf weaning weight. However, this might explain why our QTL detection for US highlighted this region as a major QTL.

The common region between US and WWm on chromosome 8 contained the *RGP1* gene which was very close (0.02 Mb) to the small QTL region shared by the three traits including the MY peak (Table 3). The *RGP1* gene (Table 4) is involved in converting guanosine diphosphate (GDP) into guanosine triphosphate (GTP) in relation to milk production [46]. On chromosome 28, the common QTL region for US and MY (Table 3) contained numerous genes (19), and we could not suggest any obvious candidates in this region, which had previously been detected as affecting MY in Brown Swiss cows [47]. On chromosome 20, the *TRIO* gene was included in the common QTL region for MY and WWm (Table 3); it promotes the exchange of GDP by GTP, in relation with milk production [46].

Regarding the common QTL for US and WWm, the common region detected on chromosome 18 (Table 3) did not correspond to any obvious candidate gene linked to suckling performance. The common QTL region detected for US and WWm on chromosome 19 contained the good candidate genes *KCNJ2* and *KCNJ16,* which are linked to the potassium channels, and are thus involved in a broad range of physiological responses in mammals. Kamikawa et al. [48] proposed that potassium channels might impact the secretion or preservation of ionic milk components in mice.

## Conclusion

The detection of QTL for calving and suckling traits highlighted nine and 15 chromosomal regions respectively, with major evidence for maternal performance in the Blonde d’Aquitaine beef breed. Regarding calving performance, three of the nine QTL regions detected had previously been reported in the literature. We were able to suggest nine candidate genes as affecting calving performance. Three of them (*LAP3*, *NCAPG*, *LCORL*), corresponding to the same QTL region on chromosome 6, have already been discussed in the literature as affecting calving performance due to either direct or maternal effects in beef breeds. In addition, six new candidate genes (*FOXG1*, *NRG1*, *SLC16A9*, *ARSJ*, and *MYH11*/*NED1*) in five different regions were associated with calving performance. In terms of suckling performance, six of the 15 QTL regions had previously been confirmed in the literature. We were able to propose seven candidate genes corresponding to six QTL regions. Two of these genes (*GC* and *ANXA7)* had also been reported in the literature as being linked to dairy cattle milk traits. Moreover, we can propose five genes (*RGP1*, *TRIO*, *ANXA7*, *KCNJ2*, *KCNJ16*) as being new candidate genes for suckling performance in beef cows.

All these candidate genes remain hypothetical because the causal mutation may be located in a regulatory region or an unmapped gene. Further studies based on sequence data will enable identification of the causal mutations underlying the 24 QTL highlighted as being linked to the maternal performance of beef cattle.

## Abbreviations

*ANXA7,* Annexin A7; *ARSJ,* Arylsulfatase Family, Member J; BF, Bayes Factor; BLUP, Best linear unbiased predictor; CS, Calving difficulty score; CSm, Maternal effect on calving difficulty score; DEBV, Deregressed Estimated Breeding Values; EBV, Estimated Breeding Values; *FOXG1*, Forkhead Box G1; *GC*, Group-Specific Component; GDP, converting guanosine diphosphate; GTP, guanosine triphosphate; HD, High density; *KCNJ16*, Potassium channel, inwardly rectifying subfamily J, member 16; *KCNJ2*, Potassium channel, inwardly rectifying subfamily J, member 2; *LAP3*, Leucine Aminopeptidase 3; *LCORL*, Ligand Dependent Nuclear Receptor Corepressor-Like; Mb, Megabase; MD, Middle density; MY, Milk yield; *MYH11*, Myosin 11; *NCAPG*, Non-SMC Condensin I Complex, Subunit G; *NDE1*, nudE Neurodevelopment Protein 1; *NPFFR2*, Neuropeptide FF Receptor 2; *NRG1*, Neuroregulin 1; PO, Pelvic opening; QTL, quantitative trait locus; *RGP1*, Retrograde Golgi Transport Homolog; *SLC11A2*, Solute carrier family 11, member 2; *SLC16A9*, Solute carrier family 16, member 9; SNP, Single nucleotide polymorphism; *TRIO*, Trio Rho Guanine Nucleotide Exchange Factor; US, Udder swelling score; WWm, Maternal effect on weaning weight.

## Additional files

Additional file 1: Number of genotyped animals in Blonde d’Aquitaine breed according to the chip density. (PNG 601 kb) Additional file 2: Strong evidence QTL for CS, PO, CSm, MY, S and WWm. (PDF 1422 kb)
