# Supplementary figures and images for: Detection of quantitative trait loci for maternal traits using high-density genotypes of Blonde d’Aquitaine beef cattle
Source: BMC Genet. 2016 Jun 21;17:88. doi: 10.1186/s12863-016-0397-y (PMC4915167; doi:10.1186/s12863-016-0397-y)

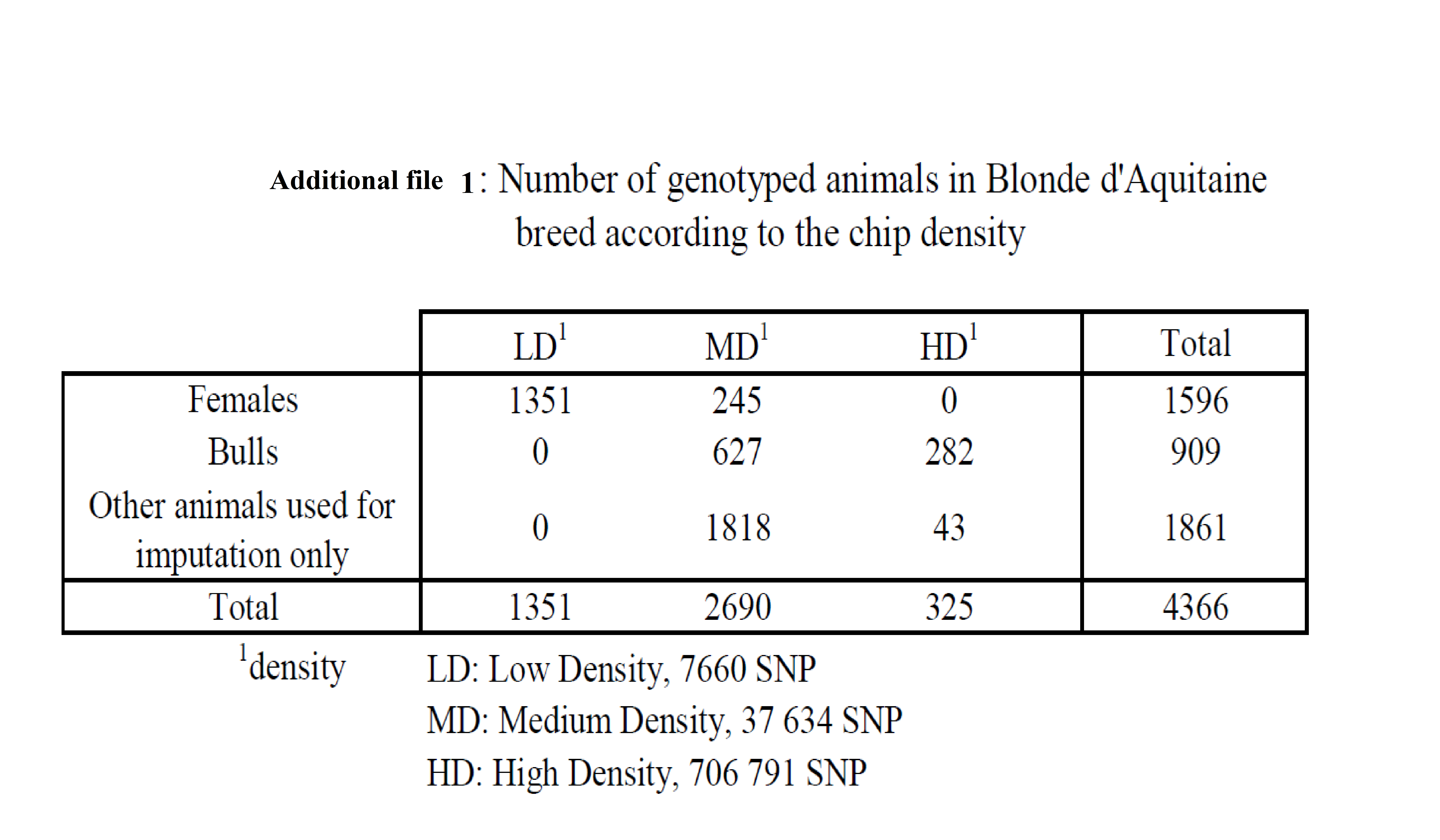

Supplement: Additional file 1: — Number of genotyped animals in Blonde d’Aquitaine breed according to the chip density. (PNG 601 kb) [file 12863_2016_397_MOESM1_ESM.png]
