# Supplementary material for: Detection of quantitative trait loci for maternal traits using high-density genotypes of Blonde d’Aquitaine beef cattle
Source: BMC Genet. 2016 Jun 21;17:88. doi: 10.1186/s12863-016-0397-y (PMC4915167; doi:10.1186/s12863-016-0397-y)
Supplement: Additional file 2: — Strong evidence QTL for CS, PO, CSm, MY, S and WWm. (PDF 1422 kb) [file 12863_2016_397_MOESM2_ESM.pdf]

Additional file 2: Strong evidence QTL for CS, PO, CSm, MY, US and WWm[1]

Trait[1]	Chromosome	Beginning (Mb)	End (Mb)	Peak Position (Mb)	Peak logBF[2]
CS	1	0.413044	0.883895	0.575063	8.3
CS	1	138.972931	140.710492	140.209951	11.3
CS	2	133.479028	134.515472	133.920047	8.6
CS	2	135.660618	136.525277	136.113224	10
CS	3	82.51374	82.680645	82.51374	8
CS	4	99.734771	100.352674	100.352674	8
CS	5	110.833453	112.208006	111.753595	8
CS	6	12.044622	12.937309	12.391211	8.4
CS	6	26.293507	26.767978	26.71183	9
CS	6	40.365425	41.46746	40.613326	8
CS	7	75.210649	75.634401	75.403195	8
CS	8	58.091386	58.599306	58.543283	10.5
CS	8	60.300234	60.344752	60.309844	8.4
CS	8	63.993433	64.438267	64.028401	11
CS	9	53.266054	53.862308	53.292843	9.6
CS	10	2.102606	2.125168	2.123404	10.7
CS	10	97.691005	100.266313	99.162103	9.3
CS	11	105.844808	106.799888	106.317253	8
CS	12	7.550418	8.153991	8.04773	8.5
CS	12	33.069158	33.117227	33.112114	10.3
CS	13	27.371883	28.377129	27.637827	8.5
CS	13	35.32933	35.767513	35.767513	8.7
CS	13	51.402282	51.981464	51.7257	8.4
CS	14	47.748256	50.273687	49.842351	8.8
CS	15	26.100483	26.11473	26.113696	10.1
CS	15	44.997531	46.675064	46.192397	8.1
CS	15	81.636633	82.683541	81.64682	11.3
CS	18	60.225621	60.993719	60.710597	8.5
CS	18	62.823945	63.42312	63.418874	8.2
CS	18	64.164125	65.604707	64.164125	9
CS	19	60.053173	60.140763	60.140763	9.7
CS	20	3.851847	4.156325	3.851847	11
CS	20	9.910852	10.590171	10.295382	10
CS	20	29.31018	29.563736	29.563736	10.2
CS	20	34.328543	35.020832	34.983531	8
CS	21	39.49298	40.269429	39.668783	12.9
CS	25	13.798454	14.529516	14.223691	10.1
CS	26	13.790152	14.130149	14.080437	8.4
CS	26	41.391613	41.708634	41.613467	8.1
CS	27	27.503845	27.877165	27.81359	16.3
CS	29	7.359818	8.175949	7.736163	8
PO	1	28.444504	28.9573	28.79087	8
PO	1	52.759474	54.033165	53.350788	8.6
PO	2	14.123835	15.820166	15.173898	11.2
PO	3	51.609263	51.92599	51.828643	9
PO	4	49.034814	52.719982	51.638897	11.4
PO	5	106.888999	108.627378	107.040157	8.8
PO	6	38.287398	39.551986	38.955154	12.5
PO	7	29.663547	31.162667	29.822944	9.9
PO	7	57.469371	57.559898	57.559898	10.4
PO	7	78.29173	78.682937	78.55706	8.3
PO	9	64.025047	64.507813	64.250148	11
PO	11	98.407974	101.454199	100.912895	8.7
PO	12	19.748215	19.748215	19.748215	8.1
PO	12	50.505079	50.659243	50.598684	8.7
PO	15	65.891394	66.496132	65.899598	9.3

Trait[1]	Chromosome	Beginning (Mb)	End (Mb)	Peak Position (Mb)	Peak logBF[2]
PO	16	43.800738	43.800738	43.800738	8
PO	19	14.299381	16.964911	14.683157	8.3
PO	19	59.893663	60.369264	60.369264	8.5
PO	19	62.859749	63.166388	62.859749	10
PO	22	27.694811	28.246185	28.034346	9.4
PO	26	18.222674	18.289903	18.252961	8.6
PO	26	42.579324	42.76507	42.579324	10.7
PO	27	11.375382	11.487379	11.487379	8
PO	28	12.666761	14.396006	14.298807	9.5
PO	28	32.158186	33.251253	33.251253	8
PO	28	33.789259	34.837468	33.892743	8
PO	28	35.437231	35.833204	35.574089	9.4
PO	28	36.442684	37.87324	37.622688	8
PO	29	42.315126	43.726898	43.466342	11.9
CSm	1	0.176459	6.475314	3.40862	8.1
CSm	3	74.380725	76.080663	74.592572	8.2
CSm	4	99.574406	100.352674	99.838409	10
CSm	5	49.553247	51.015255	50.952073	8.6
CSm	5	98.144559	101.29814	99.399578	8
CSm	6	12.391211	16.128841	15.164578	9.5
CSm	7	19.620769	21.973708	21.343266	8
CSm	7	41.420265	41.979048	41.743972	8.1
CSm	8	92.459537	96.62525	96.115837	8.2
CSm	8	99.409533	100.668219	99.822577	11.5
CSm	8	105.145819	107.411042	107.349092	8.1
CSm	8	111.713971	112.148203	111.741508	10.5
CSm	9	12.183945	13.823203	13.336308	8.8
CSm	9	21.896689	24.676123	23.665995	9.1
CSm	11	70.278649	73.525057	72.949696	12.2
CSm	11	95.146351	96.005912	95.779342	10
CSm	12	15.757561	17.392168	16.085118	8.2
CSm	13	81.012724	83.259602	81.013863	10.6
CSm	14	21.662972	26.700286	22.787711	9.2
CSm	14	53.7543	56.652113	55.567641	8.1
CSm	16	47.172573	47.608664	47.558131	8.5
CSm	16	48.342599	55.862677	54.161294	11.4
CSm	17	5.011597	7.581889	5.413848	9.1
CSm	17	28.521745	28.816998	28.696886	8.2
CSm	18	61.95172	62.363758	62.320055	8.3
CSm	25	13.798454	16.256587	16.085565	8.2
CSm	25	22.056384	23.0874	22.471137	8.1
CSm	25	34.701748	39.983243	34.701748	8.2
CSm	27	5.372349	5.373626	5.373626	8.5
CSm	27	27.775493	28.372975	27.790463	8.2
CSm	27	30.834647	33.566115	31.323447	8.5
CSm	28	15.064494	15.530655	15.458787	13.6
CSm	28	16.292217	16.759508	16.740456	8
CSm	29	18.54724	19.98825	19.003267	9.4
CSm	29	48.745169	48.959603	48.745169	8.4
MY	1	6.243987	6.809918	6.794963	11.4
MY	3	5.341039	5.367104	5.348546	8
MY	3	27.530427	28.194482	27.842537	8.3
MY	3	50.798385	51.453282	51.39675	8.3
MY	3	99.556994	102.516983	102.312083	11.4
MY	3	106.930563	107.404279	107.397431	10.6
MY	4	44.176957	46.056538	44.198598	9.6
MY	5	28.577284	29.137367	29.072132	13.4
MY	5	50.632612	51.148312	51.098866	10.6

Trait[1]	Chromosome	Beginning (Mb)	End (Mb)	Peak Position (Mb)	Peak logBF[2]
MY	5	92.425345	92.884266	92.43677	10.3
MY	6	88.485244	89.223104	88.919352	14.4
MY	6	112.334324	114.436483	114.18959	8.9
MY	7	11.241576	11.468437	11.402583	9.3
MY	7	34.25784	35.250175	35.245488	10.8
MY	7	46.495752	46.592966	46.533527	8.3
MY	7	81.180619	82.54526	81.55117	10
MY	8	60.347707	60.352572	60.352572	9.3
MY	8	85.693641	86.103145	85.773054	9.8
MY	9	46.260637	46.44134	46.390718	9
MY	10	9.1568	10.043511	9.344732	8.8
MY	10	69.747426	72.70477	70.306697	12.8
MY	10	83.403981	83.416572	83.408957	8.6
MY	11	31.437834	32.069231	32.064622	8
MY	11	79.876866	81.737824	81.453263	9
MY	13	6.909325	7.395876	6.998031	8.3
MY	13	40.511875	41.835842	41.385546	8
MY	13	44.361225	44.854912	44.84204	10.3
MY	13	50.233907	51.328106	50.33974	9
MY	13	67.431061	69.061884	67.877756	9
MY	13	82.728006	84.012692	83.805618	12.1
MY	16	74.330794	75.570408	74.368085	10.7
MY	17	4.96189	5.629449	5.049728	10
MY	17	14.147211	15.078183	14.192449	11.7
MY	17	21.729621	22.889724	22.50163	8.6
MY	19	34.262407	35.082696	34.715747	8.6
MY	19	39.196404	39.196404	39.196404	9.5
MY	19	51.451866	52.367906	51.728815	9
MY	20	1.557303	3.22742	3.199731	9.8
MY	20	3.860503	7.326646	5.504819	13.2
MY	20	18.845731	19.346879	18.895255	9.2
MY	20	54.031081	54.448092	54.041995	9.2
MY	20	57.34962	59.101797	58.162729	8.9
MY	21	67.200763	67.24838	67.200763	8.3
MY	22	26.741257	26.882476	26.741257	9.1
MY	22	55.15289	55.381125	55.201339	10.6
MY	23	21.591358	21.837732	21.620653	9.3
MY	25	25.330978	25.775647	25.419718	8.2
MY	26	21.487341	22.001261	21.759596	8.3
MY	26	29.666282	29.786019	29.782668	10.7
MY	26	36.787594	37.466186	37.435877	9.9
MY	27	29.570393	31.059321	30.229318	9
MY	27	42.374546	43.265791	42.896895	13.4
MY	28	9.141281	9.390237	9.141281	9
MY	28	43.511254	44.629957	44.036312	8.4
MY	29	47.003656	48.658082	47.805682	8.4
MY	29	50.512968	51.479989	51.478132	9.5
US	1	98.865328	100.340376	99.481997	9.3
US	4	3.827792	6.359338	4.98963	9.8
US	4	11.02856	11.903487	11.141639	9.6
US	4	43.748102	44.917034	44.260073	9.1
US	4	73.409188	73.71726	73.689211	9.9
US	4	82.747391	83.445914	83.429553	9.7
US	5	42.810659	42.810659	42.810659	8.1
US	5	88.643115	90.198826	90.198826	8
US	6	30.805204	31.600938	30.864559	8.8
US	6	52.671512	52.977553	52.814243	8.1
US	6	62.809741	63.10693	62.989278	8.3

Trait[1]	Chromosome	Beginning (Mb)	End (Mb)	Peak Position (Mb)	Peak logBF[2]
US	6	63.66279	67.173574	65.495791	9
US	6	76.278368	77.231649	77.186116	9
US	6	88.485244	88.958861	88.922396	16.3
US	6	106.5548	108.56905	107.988416	10.5
US	6	118.182364	118.345184	118.279219	9.3
US	8	16.524989	17.067907	17.052123	8.8
US	8	34.422912	36.679699	35.541388	11.5
US	8	41.845606	43.980887	43.341372	10.7
US	8	44.927523	45.230054	45.226405	8.2
US	8	60.29568	61.521362	61.044151	8.4
US	8	62.878834	64.611078	63.9871	8.5
US	8	80.269349	80.285745	80.279932	8.7
US	8	97.764033	98.625141	97.789154	8.8
US	10	4.083842	4.899211	4.868248	9.9
US	10	51.343859	52.93355	52.112302	9.1
US	10	75.68252	76.267728	76.0764	8.5
US	10	91.826482	92.561763	91.826482	9
US	10	95.851293	98.294956	96.680815	9.8
US	11	37.34907	39.059645	37.402535	8.2
US	11	104.839895	104.839895	104.839895	9
US	12	31.112988	31.838427	31.555734	9
US	12	43.637148	44.159541	44.122744	8
US	12	45.280864	45.705138	45.311592	8.7
US	12	46.989645	47.795585	46.989645	9
US	12	80.782343	81.441811	80.927587	9.5
US	13	53.21419	53.616683	53.294403	8.6
US	13	58.378178	60.922576	60.896947	8.7
US	14	12.598172	12.837793	12.694203	8.4
US	14	65.751278	65.862469	65.806497	8.4
US	15	29.736864	31.903501	30.019086	9.2
US	15	40.892563	41.339715	41.017824	8.8
US	17	54.374675	55.967061	55.221058	8.3
US	18	33.562229	34.627557	34.538807	8.9
US	19	32.295036	37.196626	34.510537	8
US	19	61.166436	61.845409	61.534509	8.2
US	21	15.301058	15.912829	15.853696	11.2
US	22	23.869047	25.888725	24.070807	9
US	22	57.454096	58.423307	57.800571	11.2
US	23	14.457482	15.614367	14.463927	10.1
US	23	17.060486	17.907638	17.632524	9.5
US	23	37.361093	37.52505	37.380904	10.2
US	24	40.441543	40.802293	40.802293	9.8
US	24	52.399133	52.905635	52.452415	8.4
US	25	18.848882	19.309494	19.276562	8.8
US	25	23.738071	24.467664	24.291733	9.1
US	26	28.000213	29.412841	28.327618	9.1
US	28	42.675311	45.870946	43.242413	9
US	29	36.914132	37.504871	37.459691	8.5
WWm	2	22.750919	24.18488	23.461494	8.6
WWm	2	66.438411	69.973324	66.9533	8.9
WWm	3	60.193295	61.066402	60.929295	8.9
WWm	3	117.005567	121.128372	118.470387	11.7
WWm	4	12.085242	21.755012	15.523374	8.8
WWm	6	6.702739	13.793449	12.523028	8.5
WWm	6	88.272818	89.494392	88.958116	8.3
WWm	6	92.437426	95.642056	93.739872	8.9
WWm	7	0.088847	3.18746	0.975609	9.5
WWm	7	3.783103	6.385558	6.29637	9.7

Trait[1]	Chromosome	Beginning (Mb)	End (Mb)	Peak Position (Mb)	Peak logBF[2]
WWm	7	24.792587	25.721686	25.00492	13.9
WWm	7	31.187696	32.655349	32.605305	8
WWm	7	84.836503	85.706354	85.086312	10.6
WWm	8	56.440073	61.783946	60.762241	8.4
WWm	9	62.228748	62.996226	62.496679	9
WWm	9	80.376913	81.616437	81.130343	8.2
WWm	9	86.573306	88.07667	87.015269	8.1
WWm	9	93.8821	96.843475	94.847446	9.1
WWm	10	65.842809	66.863235	66.288911	8.1
WWm	11	65.271707	65.969037	65.678142	8.9
WWm	11	78.70053	79.08826	79.058918	9.2
WWm	12	62.894068	63.750739	63.260943	9.7
WWm	13	72.984797	73.491631	73.163672	8.1
WWm	14	5.174657	7.510657	6.685698	8.3
WWm	15	83.686976	84.778401	84.377881	10.7
WWm	16	0.637648	1.963668	1.235699	8.9
WWm	16	22.963836	26.045099	25.158415	8.7
WWm	16	26.695043	31.17085	30.294498	9.4
WWm	17	60.358132	61.051536	60.580569	9.3
WWm	17	70.97516	74.702234	73.704127	8.5
WWm	18	27.324684	28.250625	27.341166	9.6
WWm	18	33.03004	34.341813	33.031008	9.4
WWm	19	56.224455	56.95108	56.746656	8.4
WWm	19	58.027567	63.590115	60.504374	8.9
WWm	20	3.127137	4.761369	4.126221	8
WWm	20	5.55367	8.003809	6.392965	8.6
WWm	20	38.818488	42.954273	41.691409	8.1
WWm	20	56.596319	63.835232	58.801089	9.3
WWm	22	0.358622	2.348048	1.669845	8.4
WWm	22	8.023152	9.872098	9.859141	8.5
WWm	22	10.579451	11.113761	10.659148	10.4
WWm	23	7.230705	9.640744	8.127129	9.3
WWm	23	43.823084	46.997004	45.448811	8.5
WWm	24	27.303121	29.303627	28.324646	8.1
WWm	27	14.633139	15.245767	14.763704	8.3
WWm	28	19.766274	20.801365	19.92256	14.6
WWm	28	29.412458	29.7715	29.570491	12.1
WWm	28	34.907578	35.294673	35.2367	8.5
WWm	29	44.01168	46.93549	45.892964	9

[1]Traits

CS	Calving difficulty score
PO	Pelvic opening
CSm	Maternal effect on calving difficulty score
MY	Milk yield
US	Udder swelling score
WWm	Maternal effect on weaning weight

[2]logBF log Bayes Factor
